# The role of radiological surveillance in the conservative management of incidental small testicular masses: A systematic review

**DOI:** 10.1080/2090598X.2021.1885949

**Published:** 2021-02-11

**Authors:** Dominic Brown, Georgios Tsampoukas, Elenko Petkov Popov, Zaid Aldin, Mohamad Moussa, Athanasios Papatsoris, Noor N.P. Buchholz

**Affiliations:** a Department of Urology, Broomfield Hospital, Chelmsford, UK; b Department of Urology, Princess Alexandra Hospital Trust, Harlow, UK; c U-merge Ltd.^†^ (Urology for Emerging Countries), London, UK; d Department of Urology, University Hospital of Sofia, Sofia, Bulgaria; e Department of Radiology, Princess Alexandra Hospital Trust, Harlow, UK; f Department of Urology, Al-Zahraa Hospital, University Medical Center, Beirut, Lebanon; g 2nd Department of Urology, University Hospital of Athens, Athens, Greece

**Keywords:** Testicular tumour, impalpable testicular mass, incidental testicular mass, radiological surveillance

## Abstract

**Objective**: The increasing use of scrotal ultrasonography (US) for non-cancerous indications has led to greater detection of incidental, small testicular masses. Operative intervention is currently the mainstay of treatment for all testicular tumours; however, despite the low malignant potential of small, incidental masses, little is known about conservative management using radiological surveillance.

**Methods**: A systematic review using the Preferred Reporting Items for Systematic Reviews and Meta-Analyses (PRISMA) guidelines was conducted and studies meeting the inclusion criteria were reviewed for patient outcomes.

**Results**: A total of 293 patients across six studies underwent radiological surveillance for an incidental small testicular mass. Infertility was the main indication for investigation and all studies used US as the surveillance modality. A total of 37 patients (12.6%) underwent surgical exploration during follow-up, with only 10 (3.4%) found to have malignant disease at histology.

**Conclusions**: Radiological surveillance of incidental small testicular masses is safe when used for select patient groups due to the high probability of benign disease, although optimal patient selection criteria and a well-defined protocol are lacking. This approach could be considered in patients with incidental, impalpable testicular masses of ≤5 mm in diameter displaying no significant size increase or internal vascularity on US and with negative tumour markers, as the probability of malignancy in these patients is low.

## Introduction

Testicular germ cell tumours are the most common solid neoplasms in young men with an incidence of up to 10 in 100 000 [1]. The traditionally accepted management for testicular tumours is radical orchidectomy, whilst testicular-sparing surgery (TSS) is considered for specific indications such as bilateral tumours, monorchid patients, or in children where the likelihood of benign pathology is high [2,3].

In recent years, the widespread use of ultrasonography (US) for non-cancerous indications such as infertility and orchidalgia has resulted in an increase in the detection of incidental, small testicular masses (STMs) [4,5]. Whilst clinically palpable lesions are mostly found to be malignant, impalpable tumours are predominantly benign in up to 80% of cases and therefore a non-radical surgical approach seems highly justified [6]. US allows for differentiation between testicular and para-testicular lesions with significant reliability; however, its ability to distinguish between benign and malignant intratesticular lesions is far more limited [7]. Differing definitions have been described by various authors but a STM may be defined as a testicular tumour of <10 mm in diameter, which is detected by US but remains impalpable on clinical examination [8]. As the exact pathology and natural progression of these lesions is uncertain, the current recommended approach consists of inguinal surgical exploration, delivery of the testis and acquisition of a frozen section for histopathological analysis. TSS can be performed if the lesion is found to be benign, whilst a radical orchidectomy is indicated in the presence of malignancy [9].

Operative intervention is currently the mainstay of treatment for all testicular tumours, including those classified as STMs. However, little is known about managing such lesions conservatively with monitoring in the form of radiological surveillance. Some authors have attempted to provide specific recommendations on this topic, suggesting US surveillance may be appropriate in patients with incidental, impalpable STMs due to the low possibility of malignancy [10]. Surveillance using serial US would allow early detection of those lesions carrying malignant potential allowing for further investigation and management as indicated. Despite these recommendations both optimal patient selection criteria and a protocol for radiological surveillance of STMs are lacking.

In the present systematic review, we present and discuss the current literature and available evidence surrounding the role of radiological surveillance in the management of STMs.

## Methods

A systematic review of the literature following the Preferred Reporting Items for Systematic Reviews and Meta-Analyses (PRISMA) guidelines was performed in December 2019. The following databases were searched: MEDLINE (The US National Library of Medicine’s life science database); Embase; CINAHL (Cumulative Index of Nursing and Allied Health Literature); CENTRAL (Cochrane Central Register of Controlled Trials); and PubMed. The following search terms were used when searching each database: ‘incidental testicular mass’, ‘non-palpable testicular mass’, and ‘incidental testicular tumour’.

**Figure 1.**
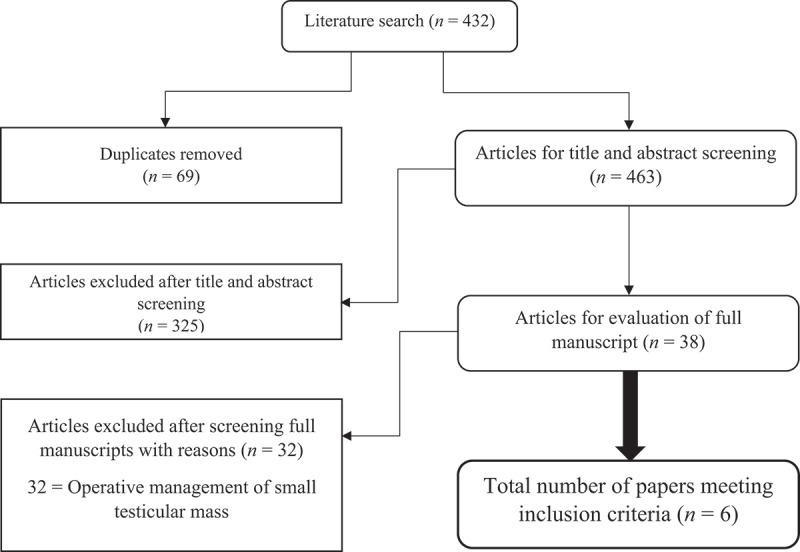
Literature flow chart demonstrating search article review and inclusion numbers

Only studies reporting radiological surveillance as the main management approach for incidental STMs in adult patients (aged >18 years) were included for review. Our search strategy is illustrated in Figure 1. The following data from each study were extracted: patient cohort demographics; main presentation, surveillance protocol and follow-up duration, tumour size, operation rate plus indication, malignancy rate/histological findings.

## Results

Six studies [11–16] were identified for inclusion in the review all of which were published in the last 14 years. All studies were retrospective except for one prospective study [11]. No randomised controlled trials comparing radiological surveillance of testicular masses to either radical orchidectomy or TSS were identified.

A total of 293 patients underwent radiological surveillance following detection of an incidental STM. The mean mass size was between 4 and 5 mm in three studies [12,13,16] and all tumours were either <10 mm [14,15] or <15 mm [11] diameter in the remaining studies.

Infertility was the main indication for US leading to the diagnosis of an incidental STM followed by scrotal or testicular pain, epididymitis, and contralateral cyst or tumour [11–16]. The dominant modality for radiological surveillance was US, whilst surveillance protocol was similar across studies consisting mostly of repeat US at 3-monthly intervals with follow-up durations ranging from 1 month to 16 years. During the follow-up period, 37 (12.6%) patients underwent explorative surgery of which 10 (3.4%) were subsequently diagnosed with malignant tumours. Surgery was performed in these cases either due to an increase in mass size or a new mass, the detection of extragonadal disease, an increase in internal vascularity, or in six cases due to patient preference [12]. All reported malignancies were germ cell tumours; predominantly seminomas with one case of embryonal carcinoma. Only two of the 10 malignant tumours were metastatic; one anaplastic seminoma, and one embryonal carcinoma. Data from the included studies are presented in Table 1 [11–16].

**Table 1.** Studies included in the review of outcomes of radiological surveillance of incidental STM lesions

Reference	No. of patients	Age, years, mean (range)	Indication for US (*n*)	Mass size, mm, mean (range)	Surveillance protocol	Follow-up, months, mean (range)	No. of operations	Operation indication (*n*)	Malignant status -Histopathology
Connolly *et al*., 2006 [16]	12	54 (34–76)	Epididymitis (5) Contralateral cyst (5) Infertility (2)	4.9 (1.5–9.8)	3-monthly scrotal US for the first year. 6-monthly scrotal US until 3 years then annually thereafter.	29.6 (6–72)	1	Increase in mass size (1)	1 malignant -1 seminoma
Toren *et al*., 2010 [12]	46	35 (21–71)	Infertility (40) Orchalgia (4) LUTS (1) Unknown (1)	4.3 (1–10)	3-monthly scrotal US	8.4 (ns)	8	Patient choice (6) Interval growth (2)	1 malignant -1 Pure seminoma 7 benign -3 Leydig cell tumour -2 Leydig cell hyperplasia -1 fibrotic nodule -1 No path. diagnosis
Isidori *et al*., 2014 [11]	27	34	Infertility (ns) Routine screening (ns) Testicular pain (ns) Contralateral tumour (ns)	<15	3-monthly scrotal US	ns (≥18)	2	Extragonadal metastases (2)	2 malignant -1 anaplastic seminoma -1 embryonal carcinoma
Bieniek *et al*., 2018 [13]	120	36.7 (23–62)	Infertility (120)	4.14 (±2 SD)	Not stated	15.6 (1.2–202.8)	18	Mass growth (ns) +ve tumour markers (ns) Patient choice (ns)	6 malignant -6 seminoma 12 benign -12 pathology ns
Li *et al*., 2017 [14]	84	42 (ns)	Scrotal pain (ns)	<10	Not stated	ns (1–84)	8	Internal vascularity (3) Size increase (1) New mass (1)	8 benign -6 Leydig cell hyperplasia -1 sarcoidosis -1 chronic inflammation
Benelli *et al*., 2017 [15]	4	30.8 (ns)	Scrotal pain (ns) Infertility (ns)	ns (2–10)	3-monthly scrotal US for the first year. 6-monthly scrotal US until 3 years then annually thereafter.	16 (ns)	0	N/A	0
Overall	293	38.75					37		10

N/A: not applicable; ns: not stated.

## Discussion

The current guidance from the European Association of Urology (EAU) on STMs states the following [17]:

- In cases of small or indeterminate testicular masses with negative tumour markers (α-fetoprotein, human chorionic gonadotropin, lactate dehydrogenase) patients should be offered TSS where possible to avoid overtreatment of potentially benign lesions and to preserve testicular function.
- Currently, there is no evidence supporting any size cut-off for a testicular lesion to be safely followed-up conservatively.
- Patients should be informed, and the physician must be aware that cancer can be found even in sub-centimetre masses therefore obtaining histology is mandatory.

The increased detection rates of small, impalpable, benign testicular masses as a result of advanced imaging techniques has raised an important question in modern urological practice regarding the optimum management of such lesions. To date there is no definitive approach and the EAU recommendations above affirm the role of operative intervention in the form of partial orchidectomy in such patients [18].

Despite the widely acknowledged likelihood of benign disease in patients undergoing routine investigation for infertility or scrotal pain with no palpable testicular mass, a consensus is yet to be reached regarding non-operative management and a well-defined, evidence-based protocol for radiological surveillance is lacking.

The acronym ‘VOMIT’ stands for Victims of Modern Imaging Technology [19] and has been used in recent years to describe patients who are over-treated for suspicious radiological findings. This phenomenon is one drawback of the increasing availability and application of sophisticated imaging systems and modalities. It illustrates the clinical dilemma of detecting an incidental small testicular lesion radiologically without being able to clearly define and predict biological progression and therefore its clinical significance. In the context of incidental testicular lesions, the acronym VOMIT can be applied to people undergoing potentially unnecessary surgical intervention in the form of orchidectomy for a clinically insignificant tumour.

Across all six studies, surgical intervention was indicated during the surveillance period in only 37 patients and only 10 of those (3.4% of total patients) were diagnosed with malignant disease. This small proportion represents the risk of having malignant disease despite having a STM deemed appropriate for radiological surveillance. Fewer patients still were diagnosed with metastatic disease (two of 293) and both findings demonstrate the relative safety of radiological surveillance in certain select cases of STM.

Infertility was the most common indication for US resulting in detection of an incidental testicular mass lesion. Despite the reported increased risk of testicular cancer in this population, the incidence of benign pathology is as high as 75% in those with incidental testicular lesions detected during infertility evaluation [20]. Patients presenting with non-obstructive azoospermia or severe semen abnormalities might demonstrate an increased risk of tumorigenesis [21], which may be associated with an underlying condition such as Klinefelter syndrome, cryptorchidism or testicular dysgenesis syndrome [22]. If surgical exploration becomes indicated in this cohort, microdissection for sperm extraction in such cases has been reported to be feasible and effective [23]. To date there is no evidence to support that obstructive azoospermia is associated with an increased risk of malignancy.

The risk of secondary hypogonadism following TSS for impalpable masses is reportedly very low in men with normal testosterone [24], although it is still a documented risk following partial orchidectomy, particularly in malignant cases [25]. This may favour an initial approach of radiological surveillance, especially in men with deteriorating endocrine function, until an indication for surgical exploration has been strongly established.

Several of the studies included in the present review highlight the role of testicular lesion size as a positive predictor for malignant disease and the subsequent need for, and degree of, surgical intervention [11–13,15]. Malignant and benign testicular lesions have been found to differ significantly in size; 12 vs 6 mm, respectively (*P* < 0.001) in one study [11], whilst larger lesions have also been found to be more likely to result in surgical intervention with one study demonstrating a significant difference in mean tumour size between operative and non-operative groups (5.38 vs 3.92 mm, respectively) [13]. Furthermore, annual tumour growth rate has been proposed as a risk factor for malignant status, with studies suggesting >0.1–0.5 mm/year growth may act as a trigger for surgical intervention [12,13].

In a separate study, 85% of incidental testicular mass lesions of <10 mm in diameter were found to remain unchanged or be benign on follow-up, whilst a cut-off of 4.5 mm was found to be an independent marker of malignancy in hypoechoic lesions [15].

Scandura *et al*. [10] collated histopathological data from 81 patients who underwent surgical exploration and reported that lesions of <5 mm in diameter in their study were always benign, whereas in comparison lesions between 5 and 10 mm were found to be malignant in one-third of cases. It is reasonable to conclude from the available evidence that a correlation can be drawn between the size of a testicular lesion and its malignant status, a factor that may play a large role in defining lesions suitable for radiological surveillance over surgical intervention.

In addition to size of the testicular mass lesion, several qualitative US features of testicular lesions have been shown to correlate with malignant status. The presence of irregular margins, microlithiasis or internal vascularisation increases the likelihood of finding malignant pathology [11]. An absence of vascularity in hypoechoic lesions favours benign disease and as such the use of colour Doppler US can better assess risk of malignancy with these tumours [13,14].

The use of multiparametric US can therefore aid diagnostic accuracy as demonstrated by Isidori *et al*. [11] who highlighted the important role of contrast-enhancement in conjunction with conventional US. The length of ‘wash-in’ and ‘wash-out’ times of contrast medium were found to be the most reliable factors when differentiating between benign and malignant disease. Another study using strain elastography (mapping the elastic properties and stiffness of soft tissue) and contrast-enhanced scrotal US in combination was able to demonstrate a sensitivity of 100%, specificity of 93% and a positive likelihood ratio of 14.3 for detecting malignancy [26].

In situations where there remains diagnostic uncertainty or multiparametric US is not available, advanced imaging in the form of MRI may be used [26,27]. MRI can demonstrate various types of lesions and tissue, distinguishing between cysts or fluid, solid lesions, fat, and fibrosis. The use of gadolinium enhancement allows the distinction between benign cystic lesions and cystic neoplasms, thus increasing diagnostic accuracy when compared to US alone [28].

Additionally, when considering the appropriateness of radiological surveillance, the possibility of a ‘burned-out’ tumour should not be overlooked. This term refers to a malignant primary testicular lesion that has undergone histological regression. These small, stable lesions may be associated with distant metastases, which may otherwise require treatment and as such raises the important issue of the need to exclude extragonadal or metastatic disease at the time of detection in patients with STMs that would otherwise be suitable for radiological surveillance [11].

As well as selecting appropriate candidates for radiological surveillance, indications for operative intervention should be well-defined. Although we are unable to make specific recommendations for these indications from the present review, it is apparent that the main indication for surgery was an increase in size of the mass during surveillance (Table 1). Mass size increase, alongside the development of extragonadal disease during the surveillance period, should therefore be considered as an indication for scrotal exploration in order to, at a minimum, obtain a histological diagnosis. Given the increasing accuracy with which modern imaging techniques are able to discern between benign and malignant disease, radiographic features of malignancy should also be given due consideration as prompts for surgical intervention if detected during a period of surveillance.

As previously discussed there exists no definitive protocol for the radiological surveillance of STM lesions; however, the majority of studies performed 3-monthly scrotal US, at least during the early stages of the observation period. In addition to the frequency of monitoring, the optimum duration of surveillance needs to be defined. In the six studies analysed in the present study, most malignant diagnoses were made early during surveillance, whilst in others the decision for surgical intervention was made because of patient preference rather than indication from surveillance.

### Implications for practice

We propose the following preliminary criteria, based on the current available literature, when defining a suitable candidate for radiological surveillance; incidentally detected testicular mass, impalpable on clinical examination, not exceeding 5 mm in diameter, demonstrating no internal vascularity on US, and no significant growth in size over an initial period of surveillance. Defining the optimum frequency and duration of US surveillance is beyond the scope of this review; however, based on previous studies, it is reasonable to perform 3-monthly interval scans in the initial surveillance period, ideally performed using multiparametric US or MRI but at a minimum with the use of colour Doppler US.

### Limitations and implications for research

Although we conducted a thorough, protocol-based systematic review of the literature it is difficult to draw evidence-based conclusions due to the few studies and limited sample size included. No randomised controlled trials comparing radiological surveillance outcomes with operative intervention were identified for inclusion and all but one study were retrospective, therefore the level of evidence available should be considered poor. Despite this, the available data reflects real-time clinical practice and as such was considered meaningful when proposing preliminary recommendations for radiological surveillance and defining suitable patient-selection criteria for further validation going forward. Additionally, we were unable to draw conclusions regarding either an optimum protocol for radiological surveillance or parameters triggering operative intervention.

The present review highlights the need for further research in this field to confirm the safety and efficacy of such a conservative approach in the management of incidental STMs. Large prospective randomised trials are needed with the aim of refining, setting-out, and validating the three following parameters:

1. Appropriate candidate selection criteria for radiological surveillance based on presentation and radiological parameters.
2. Optimum radiological surveillance protocol.
3. Patient - or radiological-related triggers for operative intervention throughout surveillance.

## Conclusion

Taking into consideration the available data from the six studies included in the present review, alongside the additional evidence presented, we conclude that radiological surveillance is a safe and appropriate management option in select cases of STMs. Further research is necessary to validate such a conservative approach if it is to become more widely accepted as a safe alternative to partial or radical orchidectomy.
